# Presepsin as a diagnostic and prognostic biomarker of severe bacterial infections and COVID-19

**DOI:** 10.1038/s41598-023-30807-5

**Published:** 2023-03-07

**Authors:** Evdoxia Kyriazopoulou, Konstantinos Leventogiannis, Georgios Tavoulareas, Efstratios Mainas, Konstantinos Toutouzas, Christos Mathas, Athanassios Prekates, Vissaria Sakka, Periklis Panagopoulos, Konstantinos Syrigos, Evangelos J. Giamarellos-Bourboulis

**Affiliations:** 1 4th Department of Internal Medicine, National and Kapodistrian University of Athens, 124 62 Athens, Greece; 2 Intensive Care Unit, Ippokrateion Athens General Hospital, 115 27 Athens, Greece; 3 1st Department of Propedeutic Surgery, National and Kapodistrian University of Athens, 115 27 Athens, Greece; 4 Intensive Care Unit, Konstantopouleio Aghia Olga General Hospital, 142 33 Nea Ionia, Greece; 5 Intensive Care Unit, Tzaneio Piraeus General Hospital, 185 36 Piraeus, Greece; 6 3rd Department of Internal Medicine, Medical School, National and Kapodistrian University of Athens, 115 27 Athens, Greece; 7 2nd Department of Internal Medicine, Medical School, Democritus University of Thrace, 681 00 Alexandroupolis, Greece; 8 4th Department of Internal Medicine, ATTIKON University General Hospital, 12462 Athens, Greece

**Keywords:** Diagnostic markers, Predictive markers, Prognostic markers, Bacterial infection, Viral infection

## Abstract

We aimed to develop presepsin as a marker of diagnosis of severe infections of either bacterial and viral origin. The derivation cohort was recruited from 173 hospitalized patients with acute pancreatitis or post-operative fever or infection suspicion aggravated by at least one sign of the quick sequential organ failure assessment (qSOFA). The first validation cohort was recruited from 57 admissions at the emergency department with at least one qSOFA sign and the second validation cohort from 115 patients with COVID-19 pneumonia. Presepsin was measured in plasma by the PATHFAST assay. Concentrations more than 350 pg/ml had sensitivity 80.2% for sepsis diagnosis in the derivation cohort (adjusted odds ratio 4.47; p < 0.0001). In the derivation cohort, sensitivity for 28-day mortality prognosis was 91.5% (adjusted odds ratio 6.82; p: 0.001). Concentrations above 350 pg/ml had sensitivity 93.3% for the diagnosis of sepsis in the first validation cohort; this was 78.3% in the second validation cohort of COVID-19 aiming at the early diagnosis of acute respiratory distress syndrome necessitating mechanical ventilation. The respective sensitivity for 28-day mortality was 85.7% and 92.3%. Presepsin may be a universal biomarker for the diagnosis of severe infections of bacterial origin and prediction of unfavorable outcome.

## Introduction

Before the COVID-19 pandemic, global epidemiology for sepsis was 49 million affected people annually leading to more than 11 million global deaths^1^. With the COVID-19 pandemic, it was realized that the epidemiology of sepsis will be seriously increased either because critical COVID-19 is a state of sepsis per se or because most of severe COVID-19 cases are complicated by bacterial sepsis^2^. In all cases, early diagnosis leading to early resuscitation is the cornerstone of treatment^3^. Unfortunately, many patients present with subtle symptoms and the use of biomarkers is the only possibility for early detection of the risk of unfavorable outcome. One major example is the approval by the European Medicines Agency of the early treatment of COVID-19 pneumonia with anakinra guided by biomarkers^4^. The rationale behind this approach is that one biomarker should not just signify the risk of unfavorable outcome but also the early stimulation of a specific mechanism which will lead to the deterioration of the human host.

CD14 is an anchored glycoprotein on the cell membranes of monocytes and macrophages. CD14 serves as a receptor of bacterial lipopolysaccharide (LPS)^5^. The soluble counterpart of CD14 is named presepsin and it is suggested to be a promising biomarker as it traceable in the circulation earlier than C-reactive protein (CRP) and procalcitonin (PCT). It is actually suggested that presepsin may increase as early as the first two hours after infection onset^6^.

We aimed to assess the diagnostic and prognostic role of presepsin for severe infections through a derivation cohort of hospitalized patients with sepsis before the start of the pandemic and two validation cohorts. The first validation cohort was recruited from patients admitted at the emergency department (ED) before the pandemic; and the second validation cohort was recruited from patients hospitalized with pneumonia due to SARS-CoV-2.

## Results

**Figure 1:**
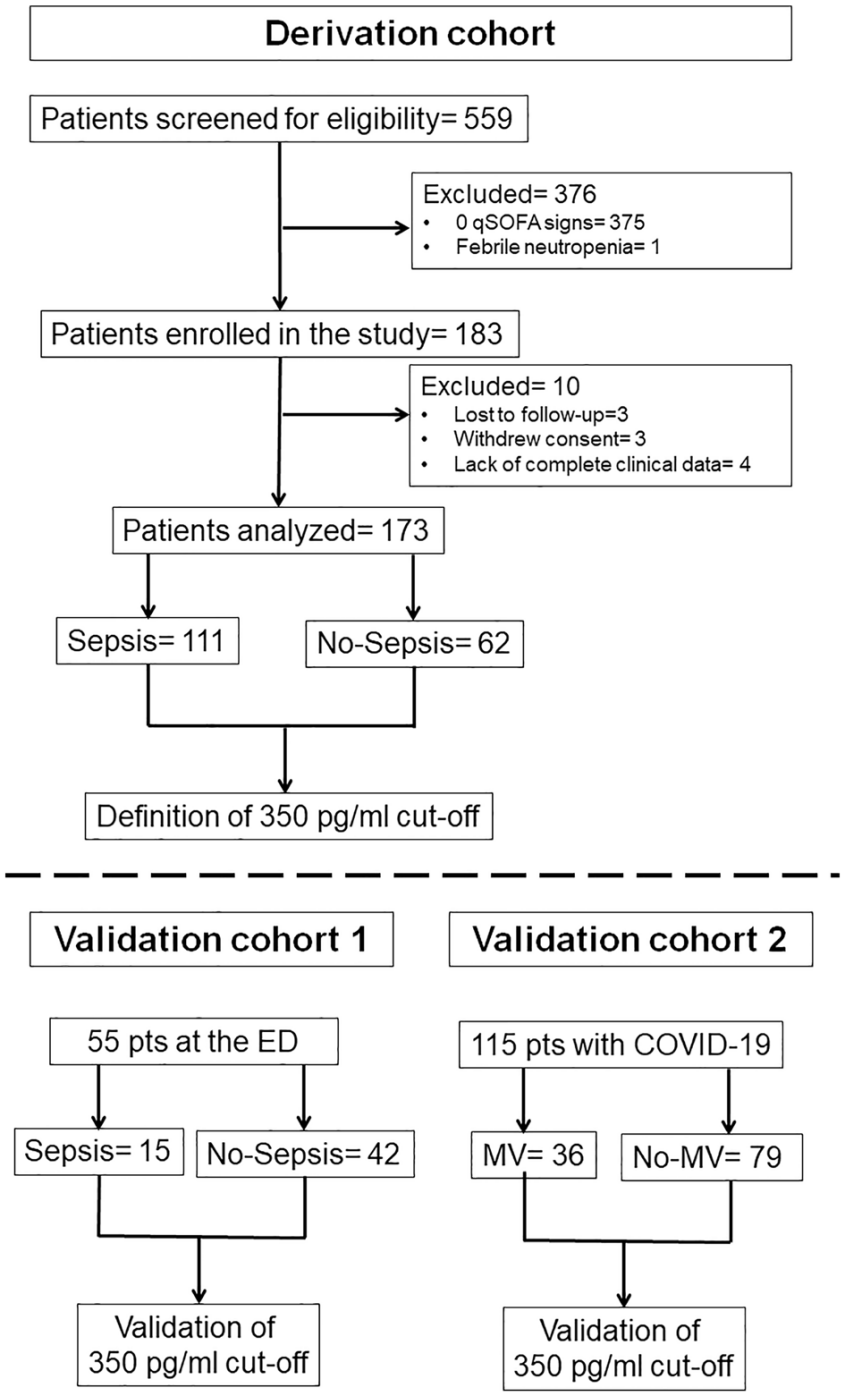
Study flowchart. The derivation cohort consisted of 173 patients recruited before the pandemic who had at least one sign of qSOFA. A cut-off of presepsin of more than 350 pg/ml was developed for the diagnosis of sepsis. This cut-off was validated in two cohorts: the validation cohort 1 was recruited from 57 consecutive admissions at the emergency department (ED) before the pandemic. The 350 pg/ml presepsin cut-off was applied for the diagnosis of sepsis the first 48 h post admission. The validation cohort 2 was recruited from 115 patients hospitalized for COVID-19 pneumonia. The 350 pg/ml presepsin cut-off was applied to demonstrate those who were in acute respiratory distress syndrome (ARDS) in need of mechanical ventilation (MV) the next 48 h.

The derivation cohort was recruited from 173 hospitalized patients who had acute pancreatitis, post-operative fever or suspicion of infection and at least one sign of the quick (q) SOFA (sequential organ failure assessment) score. The first validation cohort was recruited from 57 consecutive admissions at the emergency department (ED) of patients with suspicion of infection and at least one sign of the qSOFA score. The second validation cohort was recruited from 115 patients hospitalized for pneumonia by SARS-CoV-2 (also known as coronavirus disease-19, COVID-19) (Fig. 1, Table 1).

**Table 1:** Baseline characteristics of patients in the derivation and validation cohorts.

	Derivation cohort (n = 173)	Validation cohort 1 (n = 57)	Validation cohort 2 (n = 115)
Age, years, mean (SD)	70.9 (18.4)	72.0 (14.9)	62.0 (15.7)
Male gender, n (%)	86 (49.7)	33 (57.9)	79 (68.7)
Charlson’s Comorbidity Index, mean (SD)	4.9 (2.8)	4.4 (2.9)	2.3 (2.2)
ΑPACHE II score, mean (SD)	13.1 (7.8)	11.6 (6.4)	7.4 (4.7)
SOFA score, mean (SD)	3.7 (3.7)	2.9 (2.4)	3.3 (2.8)
On mechanical ventilation, n (%)	27 (15.6)	0 (0)	9 (7.8)
Need for vasopressors, n (%)	26 (15.0)	0 (0)	9 (7.8)
qSOFA score criteria, no. (%)			
Altered mental status	42 (24.3)	19 (33.3)	n/a
Hypotension	40 (23.1)	11 (19.3)	n/a
Tachypnea (breaths > 22/min)	151 (87.3)	36 (63.2)	n/a
White blood cells—mm^3^, mean (SD)	12,884 (7990)	10,668 (5013)	6681 (2831)
Inflammatory markers, median (Q1–Q3)			
C-reactive protein (mg/l)	65.0 (14.0–140.3)	21.3 (3.2–61.0)	64.8 (19.0–142.0)
Procalcitonin (ng/ml)	0.24 (0.06–1.31)	0.05 (0.05–0.32)	0.10 (0.05–0.34)
Presepsin (pg/ml)	763.0 (256.0–2294.0)	549.0 (312.0–1869.5)	486.0 (289.5–981.0)
Comorbidities, no (%)			
Diabetes mellitus type 2	54 (31.2)	6 (10.5)	22 (19.1)
Chronic heart failure	38 (22.0)	10 (17.5)	7 (6.1)
Chronic renal disease	28 (16.2)	9 (15.8)	3 (2.6)
Chronic obstructive pulmonary disease	31 (17.9)	10 (17.5)	9 (7.8)
Type of infection, n (%)			
Community-acquired pneumonia	72 (41.6)	10 (17.5)	115 (100)
Healthcare-associated pneumonia	16 (9.2)	0 (0)	0 (0)
Hospital-acquired pneumonia	5 (2.9)	0 (0)	0 (0)
Ventilator-associated pneumonia	3 (1.7)	0 (0)	0 (0)
Acute pyelonephritis	27 (15.6)	10 (17.5)	0 (0)
Acute biliary tract infection	11 (6.4)	1 (1.7)	0 (0)
Acute secondary peritonitis	10 (5.8)	0 (0)	0 (0)
Primary bacteremia	14 (8.1)	0 (0)	0 (0)
Intraabdominal abscess	1 (0.6)	0 (0)	0 (0)
Acute pancreatitis	4 (2.3)	0 (0)	0 (0)
Post-operative fever	11 (6.4)	0 (0)	0 (0)
SARS-CoV-2 pneumonia	0 (0)	0 (0)	115 (100.0)

*APACHE* acute physiological assessment and chronic health evaluation, *n/*a non-available, *SD* standard deviation, *SOFA* sequential organ failure assessment, *Q* quartile.

### Derivation cohort: diagnostic and prognostic performance of presepsin

**Figure 2:**
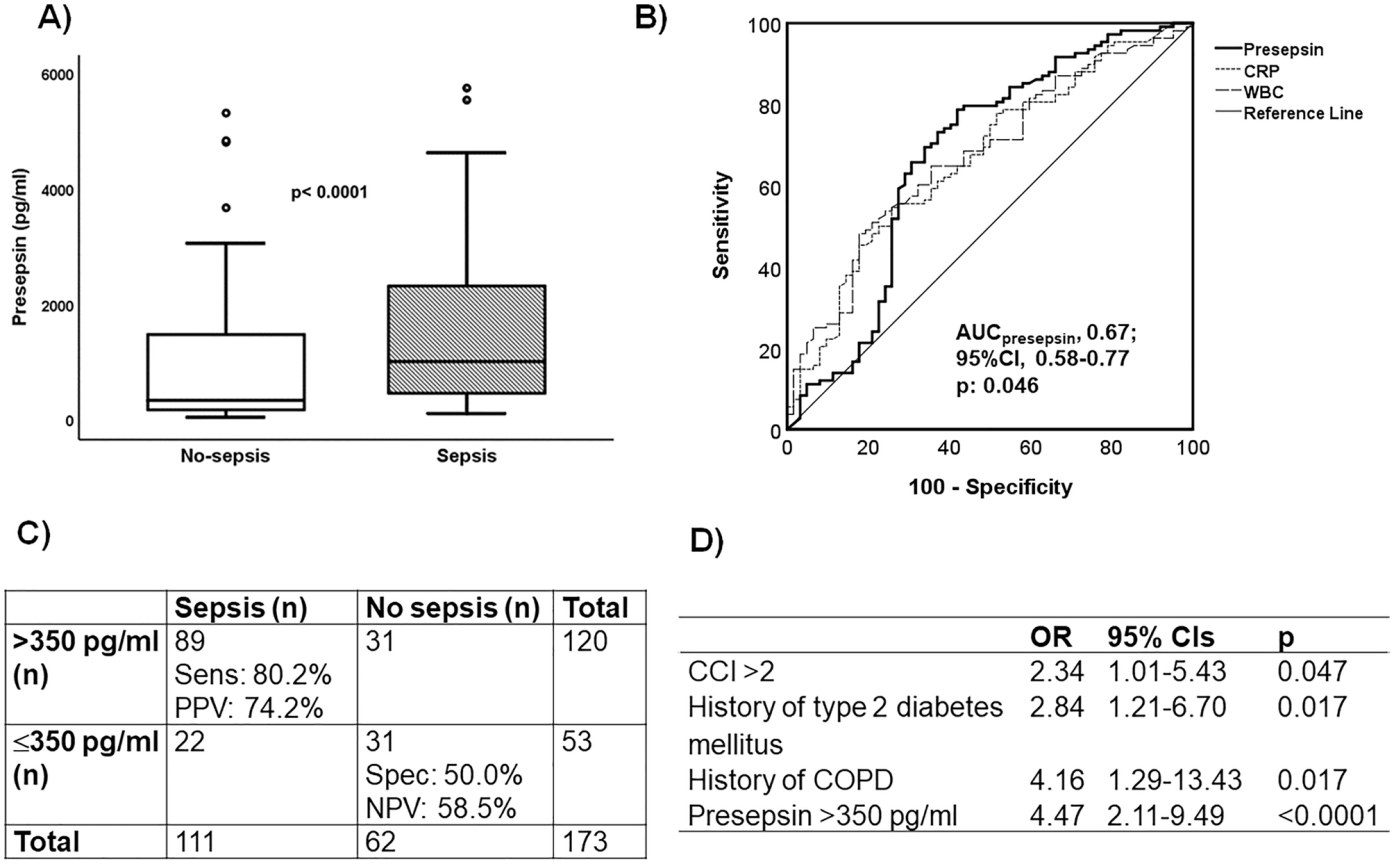
Diagnostic performance of presepsin for Sepsis using the Sepsis-3 definitions in the derivation cohort. (**A**) Box plots of the concentrations of presepsin among patients without sepsis and patients with sepsis. The p-value of the comparisons by the Mann–Whitney U test is shown. (**B**) Receiver Operator Characteristics (ROC) curve of presepsin for the diagnosis of sepsis; (**C**) diagnostic performance of the 350 pg/ml cut-off of presepsin for the diagnosis of sepsis; (**D**) stepwise (forward) logistic regression analysis of variables associated with classification into sepsis; only variables remaining significant after four steps of analysis are provided. *AUC* area under the curve, *CCI* Charlson’s comorbidity index, *CI* confidence interval, *COPD* chronic obstructive pulmonary disease, *CRP* C-reactive protein, *NPV* negative predictive value, *OR* odds ratio, *PPV* positive predictive value, *WBC* white blood cell count.

Median presepsin value of patients diagnosed with sepsis was 1024 pg/ml (Q_1_–Q_3_, 433–2345) and of those classified as non-septic 348 pg/ml (Q_1_–Q_3_,183–1548; *p* < 0.0001) (Fig. 2A). Receiver operator characteristics (ROC) curve analysis (Fig. 2B) performed in the derivation cohort showed that presepsin had better performance than white blood cell count and C-reactive protein (CRP) and a value of 350 pg/ml was the best trade-off for sensitivity and specificity. At that cut-off, sensitivity was 80.2% and specificity was 50.0% (Fig. 2C). Multivariate logistic regression analysis among all variables associated with sepsis diagnosis revealed that presepsin higher than 350 pg/ml was an independent indicator of sepsis with an adjusted odds ratio-OR 4.47 (95% CI 2.11–9.49) (Fig. 2D).

**Figure 3:**
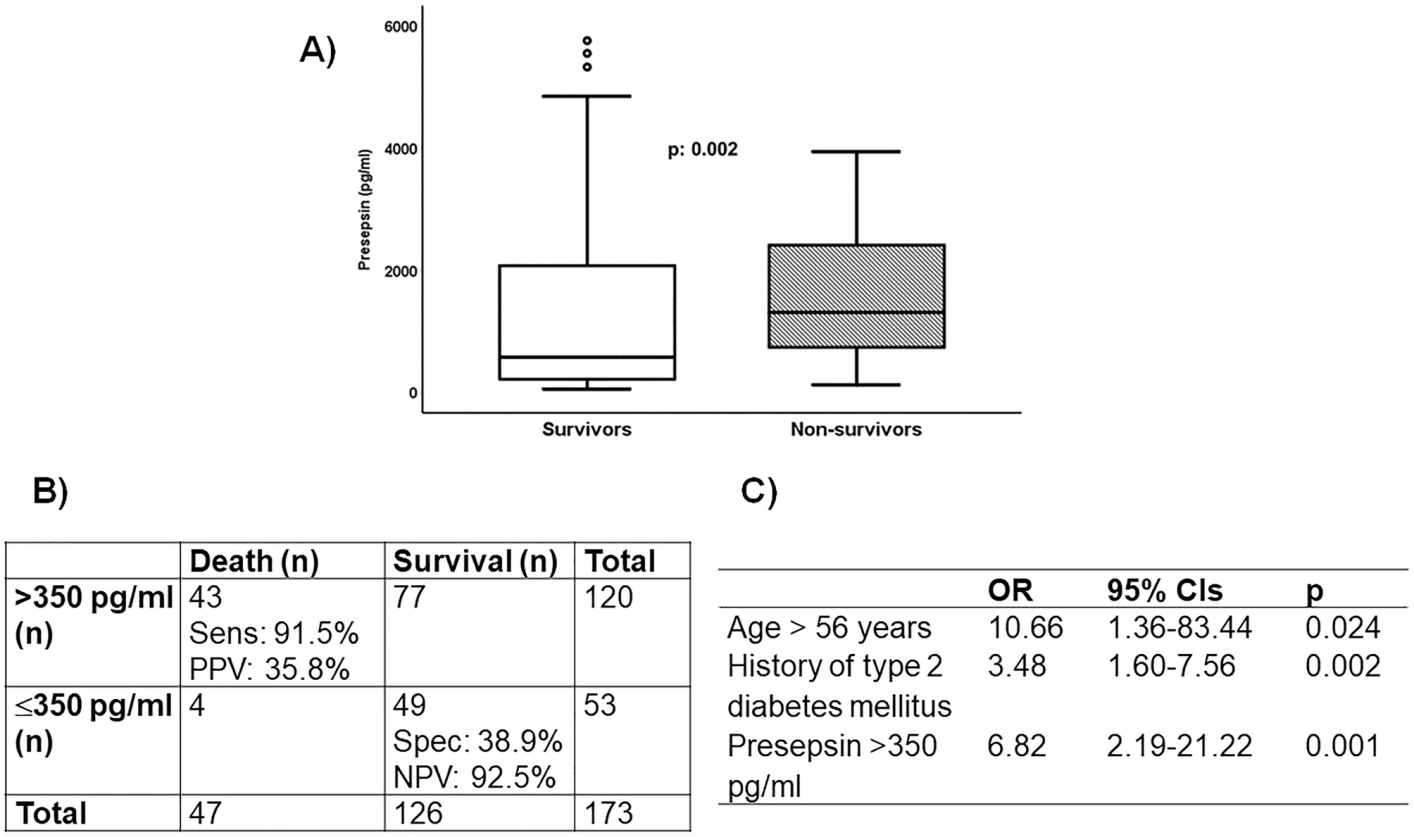
Prognostic performance of presepsin for 28-day mortality in the derivation cohort. (**A**) Box plots of the concentrations of presepsin between survivors and non-survivors. The p-value of the comparison by the Mann–Whitney U test is shown. (**B**) prognostic performance of the 350 pg/ml cut-off of presepsin for 28-day mortality; (**C**) stepwise (forward) Cox regression analysis of variables predictive of 28-day mortality; only variables remaining significant after three steps of analysis are provided. *AUC* area under the curve, *CI* confidence interval, *HR* hazard ratio, *NPV* negative predictive value, *PPV* positive predictive value.

In the derivation cohort, 47 (27.2%) patients died after 28 days. Median presepsin value of non-survivors was 1307 pg/ml (Q_1_–Q_3_, 705–2472) and of survivors 577 pg/ml (Q_1_–Q_3_, 216–2086; *p*: 0.002), respectively (Fig. 3A). When applying the cut-off of 350 pg/ml, a sensitivity as high as 91.5% was shown for the prediction of 28-day mortality (Fig. 3B). Multivariate logistic regression analysis among all variables associated with outcome revealed that presepsin higher than 350 pg/ml was an independent predictive factor of mortality with adjusted hazard ratio-HR 6.82 (95% CI 2.19–21.22) (Fig. 3C).

### Validation cohort 1: emergency department admissions

Within the validation cohort 1 of patients admitted at the ED, 41 patients had presepsin more than 350 pg/ml on hospital admission and 16 patients had presepsin 350 pg/ml or less on hospital admission. Among these patients, 14 (34.1%) and 6 (6.2%) respectively were diagnosed with sepsis the first 48 h from blood sampling (p: 0.044). Mortality after 28 days was 14.6% (6 patients) and 6.3% (one patient) respectively (p: 0.680). The diagnostic performance of presepsin more than 350 pg/ml of this validation cohort was similar for sensitivity and specificity to the derivation cohort (Supplementary Tables 1, 2).

### Validation cohort 2: COVID-19 pneumonia

Within the validation cohort 2 of patients with COVID-19 pneumonia, 71 patients had presepsin more than 350 pg/ml at hospital admission and 44 patients had presepsin 350 pg/ml or less at hospital admission. Among these patients, 29 (40.8%) and 7 (15.9%) respectively were diagnosed with acute respiratory distress syndrome (ARDS) and were in need of mechanical ventilation (MV) the first 48 h from blood sampling (OR 3.65; 95% CIs 1.43–9.31; p: 0.007). Mortality after 28 days was 16.9% (12 patients) and 2.3% (one patient) respectively (OR 8.74; 95% CIs 1.09–69.83; p: 0.016). The diagnostic and prognostic performance of presepsin more than 350 pg/ml of this validation cohort was similar to the derivation cohort (Supplementary Tables 3, 4).

## Discussion

In this study, we showed that plasma presepsin greater than 350 pg/ml has sensitivity for the diagnosis of sepsis between 80 and 90%. The biomarker performs well in both bacterial and severe COVID-19 where it has sensitivity more than 90% for the prediction of the risk of death after 28 days. In the same context, the negative predictive value of presepsin is more than 90%. The findings are developed using one derivation cohort and fully validated in two entirely different validation cohorts; one of patients admitted at the ED and another of patients with COVID-19 pneumonia.

Presepsin has been studied in several studies of bacterial sepsis and has been already compared to other biomarkers like C-reactive protein (CRP) and procalcitonin (PCT). A meta-analysis of eight studies in 2015, including a total of 1815 patients, suggested a good diagnostic performance for bacterial sepsis^7^. Two recent meta-analyses including more than 3,000 patients each, further confirmed these results^8,9^. Wu et al.^9^, estimated a pooled sensitivity of presepsin of 84% and a pooled specificity of 76% for sepsis diagnosis. Included trials suggested thresholds that varied from 317 to 849 pg/ml; although sensitivity remained constant, specificity decreased significantly when the applied thresholds exceeded 700 pg/ml.

Levels of presepsin increase in a severity-dependent manner and are strongly correlated with the development of organ failures and with severity scores like APACHE II^10^. In a cohort of 225 patients with ARDS, presepsin levels were higher in sepsis-associated ARDS than in ARDS of other etiologies and levels were also higher in non-survivors^11^.

Since presepsin is the soluble counterpart of CD14 which is associated with the toll-like receptor (TLR)-4 activation by LPS, it may be hypothesized that it is a biomarker of Gram-negative sepsis. Reported findings suggest increase in case of infection by gram-positive bacteria, fungi and viruses^12–14^. Indeed, our derivation cohort consisted by half from patients with community-acquired pneumonia mostly caused by the Gram-positive *Streptococcus pneumoniae*. Infection by the novel coronavirus-2 may represent a cause of viral sepsis in 77.9% of patients in the ICU and 33.3% of patients in the general ward^2^. To the best of our knowledge, this is the largest study so far evaluating presepsin performance in patients with COVID-19. Kocyigit et al. reported high presepsin levels in a small number of COVID-19 patients compared to healthy controls^15^.

Although bacterial co-infection or superinfection cannot be ruled out in COVID-19, the underlying mechanism of presepsin increase in COVID-19 deserves to become a matter of further research. A recent report correlates presepsin increase with neurological outcomes and survival of comatose patients after out-of-hospital cardiac arrest^16^. The ischemia and reperfusion injury may lead patients to gut ischemia and subsequent bacterial translocation similar to what is happening in sepsis^17^. This hypothesis is supported by Teixeira et al., who reported increased levels of bacterial translocation markers like LPS and CD14 in patients with COVID-19 pneumonia compared to patients with bacterial pneumonia, as well as amongst COVID-19 non-survivors and survivors^18^.

The comparable performance of presepsin in the two cohorts that differed significantly both in the source of sepsis as well as in the demographic characteristics underlines its potential benefit to be used broadly as a universal sepsis biomarker. The overtime changes of presepsin may be, like for other biomarkers such as PCT, informative about the appropriateness of the applied treatment. To this end, the lack of serial measurements in our study may be conceived as a limitation but in parallel, as a perspective for future research.

In conclusion, plasma levels of presepsin greater than 350 pg/ml detect the presence of bacterial sepsis with sensitivity exceeding 80%. Sensitivity and negative predictive value for 28-day unfavorable outcome exceed 90%.

## Methods

### Study populations

The derivation cohort included patients enrolled in the prospective observational clinical study INTELLIGENCE-1 (ClinicalTrials.gov, NCT03306186). The study was conducted in five departments (three Intensive Care Units, one department of Internal Medicine and one department of Surgery) of tertiary hospitals in Athens, Greece between October 2017 and March 2018. The INTELLIGENCE-1 study protocol was approved by the Institutional Review Boards of ATTIKON University General Hospital (approval 2089/2017); of Aghia Olga Konstantopouleion General Hospital (approval 15559/2017); of Ippokrateion Athens General Hospital (approvals 30/2017 and 47/2017); and of Tzaneio Piraeus General Hospital (approval 44/2017).

All research was performed in accordance with the Declaration of Helsinki and with relevant guidelines/regulations, and informed consent was obtained from all participants and/or their legal guardians. Inclusion criteria were: (a) adult patients of both genders; (b) patients with acute pancreatitis or post-operative fever or clinical suspicion of infection; and (c) presence of at least one sign of the quick (q) sequential organ failure assessment (SOFA) score (sudden alteration of mental status; systolic blood pressure < 100 mmHg; ≥ 22 breaths per minute). Exclusion criteria were: infection by the human immunodeficiency virus-1; less than 1000 neutrophils/mm^3^ for causes other than infection; and single trauma or multiple injuries. Written informed consent was provided from all patients or their legal representatives before enrollment. Blood was collected within the first 24 h of meeting the qSOFA criteria and patients were followed-up for 28 days. Data from medical and nursing charts were collected for demographics, comorbidities, severity indexes (acute physiology and chronic health evaluation-APACHE II and SOFA score), final diagnosis, type of infection, and 28-day mortality. The classification into sepsis or non-sepsis was done by the investigators using the Sepsis-3 definition^19^.

The first validation cohort was recruited from consecutive admissions at the ED of ATTIKON University hospital in January 2018. This cohort was given the name INTELLIGENCE-2 (approval 2455/2017 of the Institutional Review Board; Clinicaltrials.gov NCT03350113). All research was performed in accordance with the Declaration of Helsinki and with relevant guidelines/regulations, and informed consent was obtained from all participants and/or their legal guardians. Enrolled patients were adults of both genders meeting at least one of the qSOFA criteria. Exclusion criteria were: (a) known infection by the human immunodeficiency virus-1; (b) acute myocardial infarction as documented by positive electrocardiographic findings of ST-segment elevation; (c) single trauma or multiple injuries; and (d) pregnancy or lactation. Written informed consent was provided by patients or legal representatives. Blood was sampled within the first hour from ED admission.

The second validation cohort was recruited from patients hospitalized with infection by SARS-CoV-2 and screened for enrolment in the prospective trial SAVE between April and September 2020. The SAVE study was approved by the National Ethics Committee of Greece (approval 38/20) and by the National Organization for Medicines of Greece (approval IS 28/20) (ClinicalTrials.gov registration NCT04357366). All research was performed in accordance with the Declaration of Helsinki and with relevant guidelines/regulations, and informed consent was obtained from all participants and/or their legal guardians. Inclusion criteria were: (a) adults (age ≥ 18 years) of both genders; (b) molecular detection of SARS-CoV-2 by RT-PCR in nasopharyngeal samples; (c) radiological signs compatible with lower respiratory tract infection in chest X-ray or chest computed tomography^20^. Only participants failing screening or participants as comparators who did not receive intervention were analyzed in the current study. Main exclusion criteria were: infection by the human immunodeficiency virus-1; neutropenia with an absolute neutrophil count < 1500 neutrophils/mm^3^; and respiratory ratio less than 150 necessitating non-invasive or invasive ventilation. Written informed consent was provided from patients or legal representatives before enrollment. Blood was collected at hospital admission for presepsin measurement. All patients were followed-up for 28 days. Data from medical and nursing charts were collected for demographics, comorbidities, severity indexes (APACHE II and SOFA score), need for invasive mechanical ventilation (MV) and 28-day mortality.

Presepsin was measured in blood samples after plasma preparation, using the PATHFAST assay (Mitsubishi, Japan) by one independent technician blind to clinical information. PCT was measured in plasma using the VIDAS® assay (bioMérieux, Marcy l’ Etoile, France) by one independent technician blind to clinical information.

### Study endpoints

The primary endpoint of the study was the development of a cut-off of presepsin for the diagnosis of sepsis. This cut-off was originally developed in the derivation cohort and validated in the two validation cohorts. For the validation cohort 2 of patients with COVID-19 pneumonia, the cut-off of presepsin was validated for the diagnosis of ARDS necessitating MV the first 48 h. The secondary outcome was the application of this cut-off of presepsin for the prognosis of 28-day outcome.

### Sample size calculation

The derivation cohort was powered for a cut-off providing sensitivity more than 80% with 80% power at the 5% level of significance; this calculation provided a sample size of 170 patients. The first validation cohort recruited from the ED was powered using the F statistic with 80% power at the 5% level of significance considering that almost 70% of patients meeting the inclusion criteria will have sepsis; this calculation provided a sample size of 50 patients. The second validation cohort of COVID-19 was powered with 80% power at the 5% level of significance considering that almost 50% of patients meeting the inclusion criteria will need MV; this calculation provided a sample size of 110 patients.

### Statistical analysis

Categorical data were presented as frequencies and confidence intervals (CI); continuous variables with normal distribution as mean with standard deviation (SD) and data with non-normal distribution as boxplots or quartiles (Q). Fisher’s exact test was used for comparison of categorical data whereas Student’s t-test or non-parametric Mann–Whitney test were used for the comparison of continuous data, as appropriate. The diagnostic or prognostic capacity of presepsin was evaluated by the area under the respective receiver operating characteristics (AUROC) curve and the 95% CI. The optimal cut-offs were calculated by the Youden’s index. Variables associated with diagnosis of sepsis or prognosis of mortality in the univariate analysis, entered in a multivariate logistic regression model to identify those associated independently with outcomes of interest, expressed with adjusted odds ratio (OR) and 95% CI. Survival was compared by Cox analysis expressed as hazard ratio (HR) and respective 95% CI. Any two-sided *p* value lower than 0.05 was considered statistically significant. Statistical analysis was performed using the software SPSS version 25.0.

### Ethics approval and consent to participate

Written informed consent was provided from all participants or their legal representatives. The derivation cohort resulted from the observational clinical study INTELLIGENCE-1 (ClinicalTrials.gov, NCT03306186). The INTELLIGENCE-1 study was approved by the following Institutional Review Boards: ATTIKON University General Hospital, approval 2089/2017; Aghia Olga Konstantopouleion General Hospital, approval 15559/2017; Ippokrateion Athens General Hospital, approvals 30/2017 and 47/2017; Tzaneio Piraeus General Hospital, approval 44/2017. The INTELLIGENCE-2 study was approved by the Ethics Review Board of ATTIKON University General Hospital, approval 2455/2017 (Clinicaltrials.gov NCT03350113). The SAVE study was approved by the National Ethics Committee of Greece (approval 38/20) and by the National Organization for Medicines of Greece (approval IS 28/20).

## Supplementary Information

Supplementary Information.

## Data Availability

The datasets used and/or analyzed during the current study are available from the corresponding author upon reasonable request.

## References

[1] Rudd KE (2000). Global, regional, and national sepsis incidence and mortality, 1990–2017: Analysis for the global burden of disease study. Lancet.

[2] Karakike E (2021). Coronavirus disease 2019 as cause of viral sepsis: A systematic review and meta-analysis. Crit. Care Med..

[3] Kumar A (2006). Duration of hypotension before initiation of effective antimicrobial therapy is the critical determinant of survival in human septic shock. Crit. Care Med..

[4] Kyriazopoulou E (2021). Early treatment of COVID-19 with anakinra guided by soluble urokinase plasminogen activator receptor: A double-blind, randomized controlled phase 3 trial. Nat. Med..

[5] Shozushima T, Takahashi G, Matsumoto N, Kojika M, Okamura Y, Endo S (2011). Usefulness of presepsin (sCD14-ST) measurements as a marker for the diagnosis and severity of sepsis that satisfied diagnostic criteria of systemic inflammatory response syndrome. J. Infect. Chemother..

[6] Okamura Y, Yokoi H (2011). Development of a point-of-care assay system for measurement of presepsin (sCD14-ST). Clin. Chim. Acta.

[7] Zhang X, Liu D, Liu YN, Wang R, Xie LX (2015). The accuracy of presepsin (sCD14-ST) for the diagnosis of sepsis in adults: A meta-analysis. Crit. Care.

[8] Kondo Y, Umemura Y, Hayashida K, Hara Y, Aihara M, Yamakawa K (2019). Diagnostic value of procalcitonin and presepsin for sepsis in critically ill adult patients: A systematic review and meta-analysis. J. Intens. Care.

[9] Wu CC (2017). Comparison of diagnostic accuracy in sepsis between presepsin, procalcitonin, and C-reactive protein: A systematic review and meta-analysis. Ann. Intens. Care.

[10] Liu B, Chen YX, Yin Q, Zhao YZ, Li CS (2013). Diagnostic value and prognostic evaluation of presepsin for sepsis in an emergency department. Crit. Care.

[11] Zhao J, Tan Y, Wang L, Shi Y (2020). Discriminatory ability and prognostic evaluation of presepsin for sepsis-related acute respiratory distress syndrome. Sci. Rep..

[12] Masson S (2015). Circulating presepsin (soluble CD14 subtype) as a marker of host response in patients with severe sepsis or septic shock: Data from the multicenter, randomized ALBIOS trial. Intens. Care Med..

[13] Di Gioia M, Zanoni I (2015). Toll-like receptor co-receptors as master regulators of the immune response. Mol. Immunol..

[14] Wright SD (1995). CD14 and innate recognition of bacteria. J. Immunol..

[15] Kocyigit A (2021). Circulating furin, IL-6, and presepsin levels and disease severity in SARS-CoV-2-infected patients. Sci. Prog..

[16] Lansbury L, Lim B, Baskaran V, Lim WS (2021). Co-infections in people with COVID-19: A systematic review and meta-analysis. J. Infect..

[17] Qi Z, Zhang Q, Liu B, Shao F, Li C (2019). Presepsin as a biomarker for evaluating prognosis and early innate immune response of out-of-hospital cardiac arrest patients after return of spontaneous circulation. Crit. Care Med..

[18] Teixeira PC (2021). Increased LPS levels coexist with systemic inflammation and result in monocyte activation in severe COVID-19 patients. Intern. Immunopharmacol..

[19] Singer M (2016). The Third international consensus definitions for sepsis and septic shock (Sepsis-3). JAMA.

[20] Kyriazopoulou E (2021). An open label trial of anakinra to prevent respiratory failure in COVID-19. eLife.

