# Supplementary material for: Presepsin as a diagnostic and prognostic biomarker of severe bacterial infections and COVID-19
Source: Sci Rep. 2023 Mar 7;13:3814. doi: 10.1038/s41598-023-30807-5 (PMC9990570; doi:10.1038/s41598-023-30807-5)
Supplement: Supplementary file 1 — Supplementary Information. [file 41598_2023_30807_MOESM1_ESM.docx]

**Presepsin as a diagnostic and prognostic biomarker of severe bacterial infections and COVID-19: analysis through one derivation and two validation cohorts**

**Evdoxia Kyriazopoulou^1^, Konstantinos Leventogiannis^1^,**

**Georgios Tavoulareas^1^, Efstratios Mainas^2^, Konstantinos Toutouzas^3^,**

**Christos Mathas^4^, Athanassios Prekates^5^, Vissaria Sakka^6^,**

**Periklis Panagopoulos^7^, Konstantinos Syrigos^6^,**

**Evangelos J. Giamarellos-Bourboulis^1^**

^1^4^th^ Department of Internal Medicine, National and Kapodistrian University of Athens, 124 64 Athens, Greece;

^2^Intensive Care Unit, Ippokrateion Athens General Hospital, 115 27 Athens, Greece;

^3^1^st^ Department of Propedeutic Surgery, National and Kapodistrian University of Athens, 115 27 Athens, Greece;

^4^Intensive Care Unit, Konstantopouleio Aghia Olga General Hospital, 142 33 Nea Ionia, Greece;

^5^Intensive Care Unit, Tzaneio Piraeus General Hospital, 185 36 Piraeus, Greece

^6^3^rd^ Department of Internal Medicine, National and Kapodistrian University of Athens, Medical School; 115 27 Athens, Greece;

^7^2^nd^ Department of Internal Medicine, Democritus University of Thrace, Medical School, 681 00 Alexandroupolis, Greece

**Definitions of type of infections or of sterile inflammatory conditions**

*1.1 Acute pancreatitis* is defined by the presence of^1^:

- Typical epigastric pain radiating in the back

***plus*** ONE of the following:

- Serum amylase at least three time above the upper normal limit or
- Urine amylase at least three time above the upper normal limit or
- Radiographic signs in abdominal ultrasound compatible with acute pancreatitis or
- Radiographic signs in computed tomography of the upper abdomen compatible with acute pancreatitis

*1.2 Post-operation*: refers to patients undergoing either elective colectomy or elective replacement of abdominal aorta aneurysm or coronary-arterial by-pass surgery

*1.3 Clinical infection*

Patients may have one of the following well-characterized infections: community-acquired pneumonia, health-care associated pneumonia, hospital-acquired pneumonia, ventilator-associated pneumonia, acute pyelonephritis, acute biliary tract infection, intrabdominal abscess, acute secondary peritonitis or primary bloodstream infection. Each infection is defined by the following criteria:

1.3.1 Community-acquired pneumonia (CAP)^2^ as a patient without any history of contact with the hospital environment or with health-care facilities the last 90 days and presenting at least two of the following clinical/laboratory signs: a) dyspnea; b) purulent expectoration; c) auscultatory rales; and d) new consolidation in chest X-ray.

1.3.2 Health-care associated pneumonia (HCAP)^3^ as a patient either residing in a long-term care facility or undergoing chronic hemodialysis or hospitalized the last 30 days and who presents at least two of the following clinical/laboratory signs: a) dyspnea; b) purulent expectoration; c) auscultatory rales; and d) new consolidation in chest X-ray.

1.3.3 Hospital-acquired pneumonia (HAP)^3^ as a patient who presents at least two of the following clinical/laboratory signs: a) dyspnea; b) purulent expectoration; c) auscultatory rales; and d) new consolidation in chest X-ray. These signs should present at least 48 hours after hospital admission and infection should not be under development during hospital admission.

1.3.4 Ventilator-associated pneumonia (VAP)^4^ as a patient who meets ALL the following criteria:

- Intubation and mechanical ventilation for at least 48 hours;
- Purulent tracheobronchial secretions (TBS);
- New consolidation in chest X-ray or progression of a former infiltrate in chest X-ray; and
- Clinical pulmonary infection (CPIS) score more than 6; CPIS is calculated using the formula provided in APPENDIX I.

1.3.5 Acute pyelonephritis (AP)^5^ is defined by the presence of at least one clinical signs and one laboratory finding. Clinical signs are:

- Dysuria or frequency in urination;
- Flank pain;
- Pain induced after deep palpation of the right or left costo-vertebral angle

Laboratory findings are:

- Pyuria defined as more than 10 white blood cells per high power field of spun urine or positive leukocyte esterase in urine;
- Ultrasound findings compatible with acute pyelonephritis
- Radiological findings from computed urography compatible with acute pyelonephritis

1.3.6 Acute biliary tract infection (ABTI)^6^ Every patient who meets at least two of the following signs:

- Nausea and vomiting starting the last 24 hours
- Pain at the upper right quadrant starting the last 24 hours
- Jaundice starting the last 24 hours
- Pain at deep palpation of the upper right quadrant

AND

who has radiological findings on abdominal ultrasound or computed tomography of the upper abdomen compatible with acute biliary infection. These radiological findings may be one of the following: dilatation of the gallbladder, increased thickness of the wall of the gallbladder, exudation of the wall of the gallbladder, dilatation of the extrahepatic common bile duct or dilatation of the intrahepatic biliary tracts

1.3.7 Intrabdominal abscess (IAA)^6^ Every patient who presents with radiological findings on abdominal ultrasound or computed tomography of the abdomen typical of an intraabdominal abscess. These characteristics should be the well-definition of the dimensions of the abscess and intraabdominal location. The abscess may be localized either inside the liver parenchyma or in the kidney or within the intestines or in the pelvis.

1.3.8 Acute secondary peritonitis (ASP)^6^ Every patient who meets at least TWO of clinical signs and at least ONE the evidence signs. The clinical signs are:

- Decreased bowel sounds
- Start of abdominal pain at the last 24 hours
- Rebound abdominal tenderness

The evidence signs are:

- Radiological findings on abdominal X-ray or computed tomography of the abdomen compatible with acute secondary peritonitis showing free air in the abdominal cavity resulting from the rupture of an organ
- Findings compatible with ASP in open abdominal surgery

1.3.9 Primary bloodstream infection (BSI)^6^ Every patient who meets ALL the following signs:

- Peripheral blood culture positive for Gram-positive or Gram-negative bacteria or fungal species. Coagulase-negative *Staphylococcus* spp and skin commensals are considered contaminants unless isolated at least two times or isolated from both a peripheral vein and a central catheter and they have the same antibiogram
- Absence of any primary site of infection after an extensive patient work-out.

**Supplementary Table 1.** Comparative diagnostic performance of presepsin more than 350 pg/ml between the derivation cohort and the validation cohort 1. The diagnosis for both derivation cohorts is sepsis.

|  | **Derivation cohort** | **Validation cohort 1** | **p** |
| --- | --- | --- | --- |
| Sensitivity, % (95% CI) | 80.2 (71.3-86.9) | 93.3 (70.2-98.8) | 0.302 |
| Specificity, % (95% CI) | 50.0 (37.9-62.1) | 35.7 (22.9-50.8) | 0.164 |
| Positive Predictive Value, % (95% CI) | 74.2 (65.7-81.2) | 34.1 (21.6-49.4) | <0.0001 |
| Negative Predictive Value, % (95% CI) | 58.5 (45.1-70.7) | 93.8 (71.7-98.9) | 0.014 |

Abbreviation: CI confidence interval.

**Supplementary Table 2.** Comparative prognostic performance for 28-day mortality, of presepsin more than 350 pg/ml between the derivation cohort and the validation cohort 1.

|  | **Derivation cohort** | **Validation cohort 1** | **p** |
| --- | --- | --- | --- |
| Sensitivity, % (95% CI) | 91.5 (80.1-96.6) | 85.7 (48.7-97.4) | 0.515 |
| Specificity, % (95% CI) | 38.9 (30.8-47.6) | 30.0 (19.1-43.8) | 0.300 |
| Positive Predictive Value, % (95% CI) | 35.8 (27.8-44.7) | 14.6 (6.9-28.4) | 0.011 |
| Negative Predictive Value, % (95% CI) | 92.5 (82.1-97.0) | 93.8 (71.7-98.9) | 1.00 |

Abbreviation: CI confidence interval.

**Supplementary Table 3.** Comparative diagnostic performance of presepsin more than 350 pg/ml between the derivation cohort and the validation cohort 2. The diagnosis for the derivation cohort refers to sepsis and for the validation cohort refers to the presence of acute respiratory distress syndrome.

|  | **Derivation cohort** | **Validation cohort 2** | **p** |
| --- | --- | --- | --- |
| Sensitivity, % (95% CI) | 80.2 (71.3-86.9) | 78.3 (66.4-86.9) | 0.775 |
| Specificity, % (95% CI) | 50.0 (37.9-62.1) | 56.4 (43.3-68.6) | 0.492 |
| Positive Predictive Value, % (95% CI) | 74.2 (65.7-81.2) | 66.2 (54.6-76.1) | 0.240 |
| Negative Predictive Value, % (95% CI) | 58.5 (45.1-70.7) | 70.5 (55.8-81.8) | 0.222 |

Abbreviation: CI confidence interval.

**Supplementary Table 4.** Comparative prognostic performance for 28-day mortality, of presepsin more than 350 pg/ml between the derivation cohort and the validation cohort 2.

|  | **Derivation cohort** | **Validation cohort 2** | **p** |
| --- | --- | --- | --- |
| Sensitivity, % (95% CI) | 91.5% (80.1-96.6) | 92.3 (66.7-98.6) | 1.00 |
| Specificity, % (95% CI) | 38.9% (30.8-47.6) | 42.2 (33.0-51.9) | 0.617 |
| Positive Predictive Value, % (95% CI) | 35.8 (27.8-44.7) | 16.9 (9.9-27.3) | 0.005 |
| Negative Predictive Value, % (95% CI) | 92.5 (82.1-97.0) | 97.7 (88.2-99.6) | 1.00 |

Abbreviation: CI confidence interval.

**References**

1. Mylona V, Koussoulas V, Tzivras D, Makrygiannis E, Georgopoulou P, Koratzanis G, et al. Changes in adaptive and innate immunity in patients with acute pancreatitis and systemic inflammatory response syndrome. *Pancreatology* 2011; 11: 475-481
2. Christ-Crain M, Stolz D, Bingisser R, Müller C, Miedinger D, Huber PR, et al. Procalcitonin guidance of antibiotic therapy in community-acquired pneumonia. A randomized trial. *Am J Resp Crit Care Med* 2006; 174: 84-93
3. Chastre J, Fagon JY. Ventilator-associated pneumonia. *Am J Respir Crit Care Med* 2002, 165: 867-903
4. Rello J, Paiva JA, Baraibar J, Barcenilla F, Bodi M, Castander D, et al. International conference for the development of consensus on the diagnosis and treatment of ventilator-associated pneumonia. *Chest* 2001, 120: 955-970
5. Pinson AG, Philbrick JT, Lindbeck GH, Schorling JB. Fever in the clinical diagnosis of acute pyelonephritis. *Am J Emerg Med* 1997; 15: 148-151
6. Calandra T, Cohen J. The International Sepsis Forum Consensus definitions of infections in the intensive care unit. *Crit Care Med* 2005; 33: 1639-1648
